# MicroRNA-221 promotes cisplatin resistance in osteosarcoma cells by targeting PPP2R2A

**DOI:** 10.1042/BSR20190198

**Published:** 2019-07-10

**Authors:** Wen-chao Yu, Hui-hao Chen, Yan-yan Qu, Chun-wei Xu, Chen Yang, Yan Liu

**Affiliations:** 1 Department of Orthopedic Surgery, Changzheng Hospital, Shanghai 200003, China; 2 Center for Community Health Service, Sub-District of West Nanjing Road, Jing An District, Shanghai, China; 3 Department of Pathology, Fujian Cancer Hospital, Fujian Medical University Cancer Hospital, Fuzhou 350014, China

**Keywords:** cisplatin, microRNA-221, osteosarcoma cells, PPP2R2A

## Abstract

Osteosarcoma (OS), the most common malignant bone tumor, is the main cause of cancer-related deaths in children and young adults. Despite the combination of surgery and multi-agent chemotherapy, patients with OS who develop resistance to chemotherapy or experience recurrence have a dismal prognosis. MicroRNAs (miRNAs) are a class of small noncoding RNAs that repress their targets by binding to the 3′-UTR and/or coding sequences, leading to the inhibition of gene expression. miR-221 is found to be up-regulated in tumors when compared with their matched normal osteoblast tissues. We also observed significant miR-221 up-regulation in the OS cell lines, MG-63, SaoS-2, and U2OS, when compared with the normal osteoblast cell line, HOb. Overexpression of miR-221 promoted OS cell invasion, migration, proliferation, and cisplatin resistance. MG-63 and SaoS-2 cells transfected with miR-221 mimics were more resistant to cisplatin. The IC_50_ of MG-63 cells transfected with control mimics was 1.24 μM. However, the IC_50_ of MG-63 cells overexpressing miR-221 increased to 7.65 μM. Similar results were found in SaoS-2 cells, where the IC_50_ for cisplatin increased from 3.65 to 8.73 μM. Thus, we report that miR-221 directly targets PP2A subunit B (PPP2R2A) in OS by binding to the 3′-UTR of the PPP2R2A mRNA. Restoration of PPP2R2A in miR-221-overexpressing OS cells recovers the cisplatin sensitivity of OS cells. Therefore, the present study suggests a new therapeutic approach by inhibiting miR-221 for anti-chemoresistance in OS.

## Introduction

As the most common bone tumor, osteosarcoma (OS) causes cancer-related deaths especially in children and young adults [1]. Patients with OS showing local recurrence or metastasis often have a poor outcome [2]. Although the molecular mechanisms of OS are still poorly understood, long-term survival rates in OS have improved in the past few years [2]. Cisplatin is among the three most active chemotherapeutic drugs (methotrexate, cisplatin, and doxorubicin) used to treat OS [3]. However, the frequent development of cisplatin resistance and recurrence of additional malignancies is a serious problem that greatly impairs the efficiency of chemotherapy [4]. Therefore, finding novel biomarkers for the diagnosis, prognosis, and treatment of OS is necessary to improve the clinical treatments for patients with OS.

MicroRNAs (miRNAs) are a class of small noncoding RNAs that repress their targets by binding to the 3′-UTR, leading to the inhibition of gene expression [5,6]. They play crucial roles in regulating cell proliferation, apoptosis, angiogenesis, chemosensitivity, metabolism, and metastasis in multiple cancers including OS [6]. miR-221 has been reported to display oncogenic roles in multiple cancers such as colon cancer [7], breast cancer [8], lung cancer [9], liver cancer [10], and OS [11]. However, the molecular mechanisms are still under investigation. Therefore, identifying novel miRNAs that are involved in OS progression and chemoresistance can contribute to the development of therapeutic strategies for the diagnosis and treatment of OS.

Protein phosphatase 2 (PP2A) is a heterotrimeric serine/threonine (Ser/Thr) phosphatase that consists of one structural (PP2A/A), one catalytic (PP2A/C), and one regulatory (PP2A/B) subunit [12]. As a Ser/Thr phosphatase, PP2A participates in the dephosphorylation of Ser/Thr phosphorylation, resulting in defective or deregulated PP2A in cancer [13,14]. The PP2A/B subunit has multiple isoforms that determine substrate specificity [12]. PPP2R2A, which codes for the α isoform of the regulatory B55 subfamily of PP2A, is involved in the negative control of cancer cell growth and development [15,16]. Recent study showed that miR-222 could attenuate cisplatin-induced cell death in bladder cancer cells through inhibiting the cisplatin-induced autophagy in bladder cancer cells by directly targeting PP2A subunit B (PPP2R2A) [17], indicating miRNA could modulate cisplatin sensitivity of OS by targeting PPP2R2A. In addition, the PPP2R2A was known as an upstream negative effector of Akt/mTOR pathway [18], suggesting down-regulation of PPP2R2A could impair tumor chemosensitivity. The present study aims to identify the roles of miR-221 in OS. It will also investigate the miR-221-modulated cisplatin sensitivity of OS as well as the direct targets of miR-221 in OS.

## Materials and methods

### Cell culture and tumor specimens

Human OS cell lines MG-63 and SaoS-2 were obtained from the American Type Culture Collection (ATCC, Rockville, MD, U.S.A.) and maintained in RPMI-1640 medium (Gibco-BRL) supplemented with 10% fetal bovine serum (FBS) and 1% penicillin–streptomycin (Gibco-BRL) at 37°C in the presence of 5% CO_2_. Human osteoblast (HOb) cells (Sigma–Aldrich, St. Louis, MO, U.S.A.) were maintained in DMEM/F-12 containing 10% FBS and 1% penicillin–streptomycin at 37°C in a 5% CO_2_ humidified incubator. Cells were routinely passaged when they reached ∼80% confluence. Fifteen pairs of human OS tissues and their adjacent normal osteoblast tissues were obtained between January 2012 and January 2016 from the Department of Orthopedics, Changzheng Hospital. After surgical resection, the tissues were stored in liquid nitrogen immediately. No patient had received radiotherapy or chemotherapy prior to surgery. All tumor samples were diagnosed as high-grade OS of stage IIA or IIB, according to the Enneking staging system [19]. The mean age of the patients was 20 years (range: 17–31 years), and 61% were male. Among the IIA or IIB Enneking stage tumors, eight patients were at IIA stage and seven patients were at IIB stage. Histological subtype was five from special and ten from conventional. Two patients were with metastasis. All diagnoses were confirmed by an experienced pathologist. Written informed consent from all the patients and approval from the Institutional Research Ethics Committee of Changzheng Hospital were also obtained.

### Antibodies and reagents

Mouse monoclonal antibodies against PPP2R2A and β-actin were purchased from Cell Signaling (#5689 and #3700; Danvers, MA, U.S.A.). Cisplatin and doxorubicin were purchased from Sigma–Aldrich (Shanghai, China).

### Transfection of miRNAs and plasmid DNA

Cells were seeded into six-well plates and grown to 70–80% confluence, following which transfections were performed using the Lipofectamine™ 2000 transfection reagent (Invitrogen, U.S.A.) according to the manufacturer’s instructions. miR-221 or control mimics were transfected at 50 nM concentrations for 48 h. The miR-221 and control mimics were purchased from GenePharma Co. Ltd. (Shanghai, China). The overexpression vector that contained the wild-type PPP2R2A and lacked the 3′-UTR was constructed by Genesil Biotechnology Co. Ltd. (Wuhan, China). Plasmid DNA was transfected into cells at 2 μg for 48 h and followed by downstream assays.

### Cell viability assay

Cell viability was examined using 3-(4,5-dimethylthiazol-2-yl)-2,5-diphenyltetrazolium bromide (MTT) assay. Cells were seeded into 96-well plates at a density of 5000 cells/well with 100 μl of culture medium and incubated overnight. They were then transfected with 50 nM of negative control, miR-221 mimics, or miR-221 inhibitor for 48 h followed by cisplatin treatments at the indicated concentrations. After 24 h of treatment, 20 ml of 5 mg/ml MTT (Sigma–Aldrich) was added into each well and incubated for 4 h in a humidified incubator. After discarding the medium, 200 μl of dimethyl sulfoxide (DMSO) was added to each well to dissolve the formazan. Optical density (OD) was evaluated by measuring the absorbance at a wavelength of 490 nm.

### Caspase activity

The activity of caspase-3 was analyzed using the Caspase 3 Assay Kit (Colorimetric) (ab39401; Abcam, Cambridge, U.K.) according to the manufacturer’s instructions.

### Target prediction

Bioinformatics analysis to predict the miR-221 target was performed using the online program TargetScan (http://www.targetscan.org/).

### qRT-PCR

Total RNA was extracted from the cells or tissues using TRIzol reagent (Invitrogen) according to the manufacturer’s instructions. The quality of the RNA samples was detected using a Nanodrop™ 2000 spectrophotometer (Thermo Fisher Scientific, Waltham, MA, U.S.A.). Next, cDNA was reverse transcribed from the total RNA via specific miR-221 primers using the TaqMan™ MicroRNA Assays kit and reagents from the TaqMan™ MicroRNA Reverse Transcription Kit (Thermo Fisher Scientific) according to the manufacturer’s instructions. The real-time PCR results were normalized to the internal control, small nucleolar RNA (RNU6). The relative expression levels were calculated using the 2^−ΔΔ*C*^_t_ method. All the experiments were performed in triplicate.

### Luciferase reporter assay

The luciferase reporter assay was performed according to a previous report [20]. The human PPP2R2A 3′-UTR containing the miR-221 binding site was amplified and cloned into the pMIR-REPORT vector (Life Technologies) to generate the wild-type pMIR-PPP2R2A-3′-UTR reporter. A mutant fragment with the miR-221 target site was synthesized and cloned into the same vector to generate a mutant pMIR-PPP2R2A-3′-UTR reporter. The reporters and control or miR-221 mimics were co-transfected into MG-63 and SaoS-2 cells for 48 h using the Lipofectamine™ 2000 transfection reagent (Invitrogen). The luciferase activity was measured using the Dual-Luciferase® Reporter Assay System (Promega, Madison, WI, U.S.A.) as described previously [20]. The results were modified as relative luciferase activity (firefly Luc/*Renilla* Luc). All the experiments were performed in triplicate.

### Western blot analysis

OS cells were lysed in RIPA buffer (Thermo Fisher Scientific) containing 1× proteinase inhibitor cocktail (Thermo Fisher Scientific) while on ice for 30 min. The lysates were then centrifuged at 12000 rpm for 10 min in order to collect the supernatant as total proteins. Protein concentrations were determined using the Bradford assay. Equal amounts of protein lysates (40 μg each) were separated using 4–20% SDS/PAGE and electro-transferred to nitrocellulose membranes (Invitrogen). The membranes were blocked with TBST containing 5% non-fat dry milk for 1 h at room temperature and incubated overnight at 4°C with primary antibodies against PPP2R2A and β-actin. After washing, the membranes were incubated with horseradish peroxidase–conjugated anti-rabbit secondary antibodies (Santa Cruz, U.S.A.) at room temperature for 2 h. After washing again, the target protein was detected via chemiluminescence using the Pierce ECL Western Blotting Substrate (Thermo Fisher Scientific).

### Statistical analysis

All the data are expressed as mean ± SD, where the error bars represent the standard deviation of the mean. Student’s *t* test and one-way analysis of variance (ANOVA) were used to calculate the statistical significance. *P*<0.05 was considered to be statistically significant. Statistical analyses were performed using the SPSS 13.0 statistics software (SPSS Inc, U.S.A.).

## Results

### miR-221 is up-regulated in OS cells and tumors

**Figure 1:**
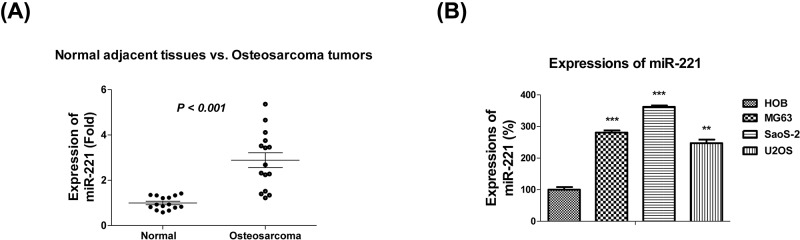
miR-221 is up-regulated in the tissues and cell lines of patients with OS (**A**) miR-221 expressions were analyzed using qRT-PCR and normalized to RNU6 in 15 OS tumors and their matched normal tissues. (**B**) miR-221 expressions were analyzed using qRT-PCR and normalized to RNU6 in three OS cell lines. miR-221 expression in normal HOb cells was used as a control. Data are presented as mean ± SD. Columns, mean of three independent experiments; bars, SD; **, *P*<0.01; ***, *P*<0.001.

Previous studies demonstrated miR-221 to be up-regulated in multiple cancers [7–11]. To elucidate the role of miR-221 in OS, we measured the expression level of miR-221 in human OS tissues and cells. We found miR-221 to be significantly up-regulated in tissue samples from patients with OS when compared with the normal adjacent tissue samples in 15 paired OS and normal tissues (Figure 1A). We also observed significant up-regulation of miR-221 in the OS cell lines, MG-63, SaoS-2, and U2OS, when compared with the normal osteoblast cell line, HOb (Figure 1B). Cumulatively, our results indicate miR-221 to be an oncogenic miRNA in OS.

### miR-221 overexpression promotes OS cell invasion, migration, and proliferation

**Figure 2:**
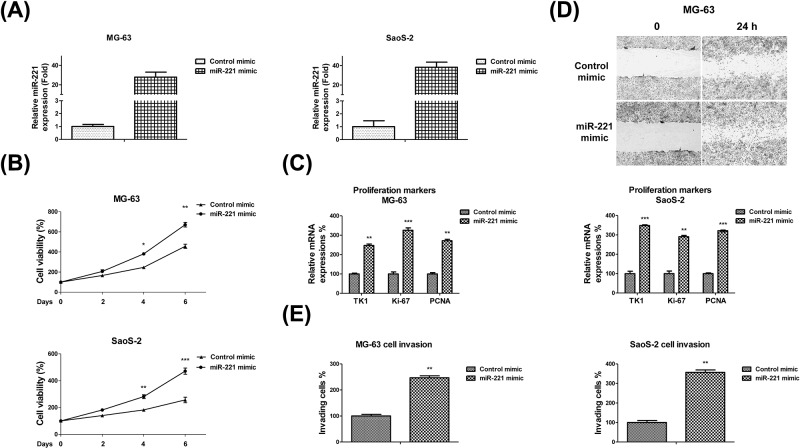
miR-221 overexpression promotes OS cell proliferation, migration, and invasion (**A**) miR-221 or control mimics were transfected into MG-63 (left) and SaoS-2 (right) cells at 50 nM concentrations for 48 h. miR-221 expressions were analyzed using qRT-PCR and normalized to RNU6. (**B**) MG-63 (upper) and SaoS-2 (lower) cells were transfected with 50 nM of control or miR-221 mimics for 48 h, followed by the measurements of cell proliferation via MTT assay and (**C**) measurements of the cell proliferation markers, TK1, Ki-67, and PCNA, using qRT-PCR. (**D**) MG-63 cells were transfected with 50 nM of control or miR-221 mimics for 48 h. Cell migration was measured via wound healing assay and (**E**) cell invasion was measured via transwell assay. Data are presented as mean ± SD. Columns, mean of three independent experiments; bars, SD; *, *P*<0.05; **, *P*<0.01; ***, *P*<0.001.

To verify whether miR-221 plays oncogenic roles in OS, we transfected miR-221 or control mimics into MG-63 and SaoS-2 cells for 48 h. miR-221 expressions in MG-63 and SaoS-2 cells transfected with miR-221 mimics increased to 36.7- and 28.8-folds respectively when compared with their control mimic-transfected cells (Figure 2A). To investigate the putative oncogenic functions of miR-221 in OS cells, we detected the proliferation rate, *in vitro* cell migration, and invasive capacity of miR-221-overexpressing OS cells. As expected, the viability significantly increased to 1.35- and 1.65-folds in MG-63 and SaoS-2 cells respectively with miR-221 overexpression when compared with the control mimic-transfected cells (Figure 2B). Moreover, we also detected the cellular proliferative markers, thymidine kinase 1 (TK1), Ki-67, and PCNA, using real-time PCR and found that their mRNA expressions were significantly up-regulated in miR-221-overexpressing OS cells (Figure 2C). By performing a classical scratch-wound healing assay, we found that the OS cells overexpressing miR-221 exhibited a higher migration and invasion rate when compared with the cells in the control group (Figure 2D). Furthermore, invasion assays using a transwell invasion chamber were performed to examine the invasive ability of MG-63 and SaoS-2 cells. We observed that a greater number of MG-63 cells overexpressing miR-221 invaded the Matrigel membrane when compared witho the control group (Figure 2E). These results indicate that miR-221 enhances the *in vitro* invasive and migration capacities of OS cells.

### miR-221 modulates the chemosensitivity of OS cells

**Figure 3:**
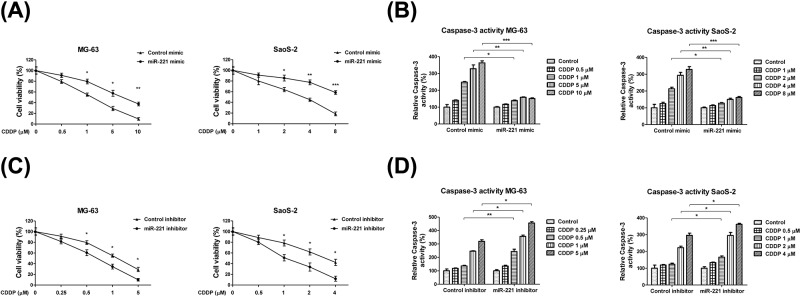
miR-221 modulates cisplatin sensitivity in OS cells (**A**) MG-63 (left) and SaoS-2 (right) cells were transfected with 50 nM of control or miR-221 mimics for 48 h. Cells were treated with the indicated cisplatin concentrations for 48 h. Cell viability was measured via MTT assay and (**B**) cell apoptosis was measured via caspase activity assay. (**C**) MG-63 (left) and SaoS-2 (right) cells were transfected with 50 nM of control or miR-221 inhibitors for 48 h. Cell viability at the indicated cisplatin concentrations was measured via MTT assay and (**D**) cell apoptosis was measured via caspase activity assay. Data are presented as mean ± SD. Columns, mean of three independent experiments; bars, SD; *, *P*<0.05; **, *P*<0.01; ***, *P*<0.001.

Cisplatin is known to exert its anti-tumor effects by inducing cancer cell apoptosis in multiple cancers [21]. To gain further insight into the oncogenic roles of miR-221 in anti-cancer agent-induced cytotoxicity, we studied the sensitivity of OS to cisplatin with or without the exogenous overexpression of miR-221. As shown in Figure 3A, MG-63 and SaoS-2 cells transfected with miR-221 mimics were observed to be more resistant to cisplatin. The IC_50_ of MG-63 transfected with control mimics was observed to be 1.24 μM. However, the IC_50_ of miR-221-overexpressing MG-63 cells was observed to increase to 7.65 μM (Figure 3A). Similar results were found in SaoS-2 cells, where the IC_50_ for cisplatin increased from 3.65 to 8.73 μM (Figure 3A). To assess whether the miR-221-modulated cisplatin resistance occurred via the inhibited cellular apoptosis pathway, we performed caspase assays to detect caspase-3 activity under cisplatin treatments. As expected, caspase-3 activity under cisplatin treatments was suppressed by miR-221 overexpression (Figure 3B). In order to analyze the effects of miR-221 inhibition on the cisplatin sensitivity of OS cells, we transfected an miR-221 inhibitor into MG-63 and SaoS-2 cells (Supplementary Figure S1). MTT assays demonstrated that miR-221 inhibition enhanced the cisplatin sensitivity of OS cells (Figure 3C). Furthermore, inhibition of miR-221 significantly elevated the caspase activity of OS cells under cisplatin treatments (Figure 3D). To investigate whether the miR-221-modulated cisplatin sensitivity was time dependent, we examined the effects of miR-221 overexpression on the cisplatin sensitivity of OS cells under cisplatin treatments at 72 and 96 h. As expected, miR-221 overexpression increased the resistance of OS cells to cisplatin at 72 and 96 h (Supplementary Figure S2A,B). However, miR-221 inhibition sensitized OS cells to cisplatin at 72 and 96 h (Supplementary Figure S2C,D). Next, we investigated whether miR-221 regulated other anti-OS drugs. Since doxorubicin is a widely used chemotherapeutic agent for treating OS [22], we assessed the sensitivity of OS cells to doxorubicin with or without miR-221 overexpression. MG-63 and SaoS-2 cells were transfected with control or miR-221 mimics for 48 h and then treated with doxorubicin at 0, 1, 5, 10, or 20 μg/ml and 0, 10, 20, 40, or 80 ng/ml, respectively. As expected, cell viability and apoptosis assays showed that miR-221 overexpression decreased the cell sensitivity to doxorubicin (Supplementary Figure S3). Collectively, these results suggested that miR-221 inhibition promoted chemotherapeutic agent-induced OS cell death.

### miR-221 directly targets the 3′-UTR of PPP2R2A

**Figure 4:**
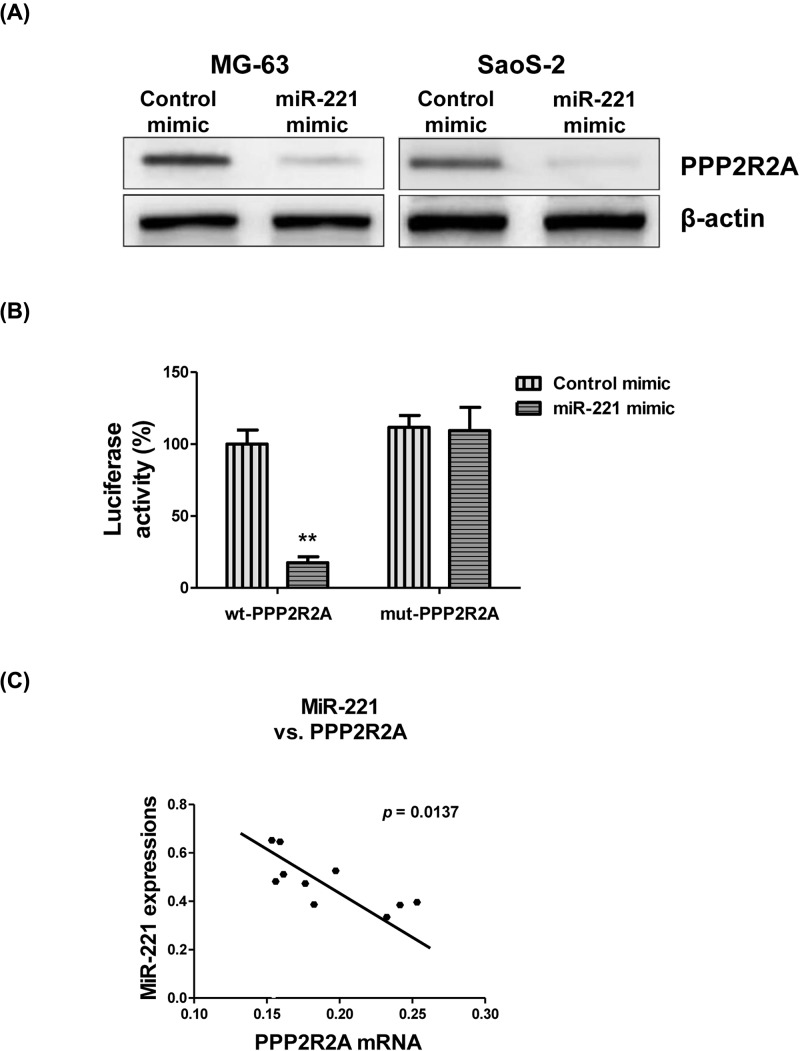
miR-221 directly targets PPP2R2A (**A**) MG-63 and SaoS-2 cells were transfected with 50 nM of control or miR-221 mimics for 48 h, followed by Western blot analysis of PPP2R2A expression. (**B**) Luciferase assay showing miR-221 binding to the 3′-UTR of PPP2R2A. (**C**) Negative correlation between miR-221 and PPP2R2A mRNA in OS tumors. Data are presented as mean ± SD. Columns, mean of three independent experiments; bars, SD; **, *P*<0.01.

Next, we investigated the molecular mechanism of miR-221-mediated cisplatin sensitivity. The potential targets of miR-221 were analyzed by searching the TargetScan database, which indicated PPP2R2A as a potential target of miR-221 (data not shown). Moreover, the binding regions in PPP2R2A were also shown to be conserved over multiple species (data not shown). Interestingly, PPP2R2A is a known target of miR-221 in hepatocellular and bladder cancer cells [23,24]. Next, we determined if PPP2R2A was indeed a target of miR-221 by transfecting MG-63 and SaoS-2 cells with 100 nM of miR-221 or control mimics. Western blot analysis revealed that PPP2R2A protein expression was considerably down-regulated by miR-221 overexpression (Figure 4A). To test whether miR-221 directly targeted the 3′-UTR of the PPP2R2A mRNA, dual-luciferase assay was performed. The results showed that miR-221 overexpression significantly decreased the luciferase activity of the vectors containing the wild-type PPP2R2A-3′-UTR, but not that of the vectors containing the mutant PPP2R2A-3′-UTR (Figure 4B), suggesting PPP2R2A to be a direct target of miR-221. Consistently, we also observed a significant negative correlation between PPP2R2A mRNA and miR-221 in samples from patients with OS (Figure 4C). The relatively high miR-221 levels expressed in the OS tumors were accompanied by low PPP2R2A mRNA expression levels. Collectively, these results indicated that PPP2R2A was a direct target of miR-221 in OS both, *in vitro* and *in vivo*.

### Restoration of PPP2R2A in miR-221-overexpressing OS cells recovers the cisplatin sensitivity of OS cells

**Figure 5:**
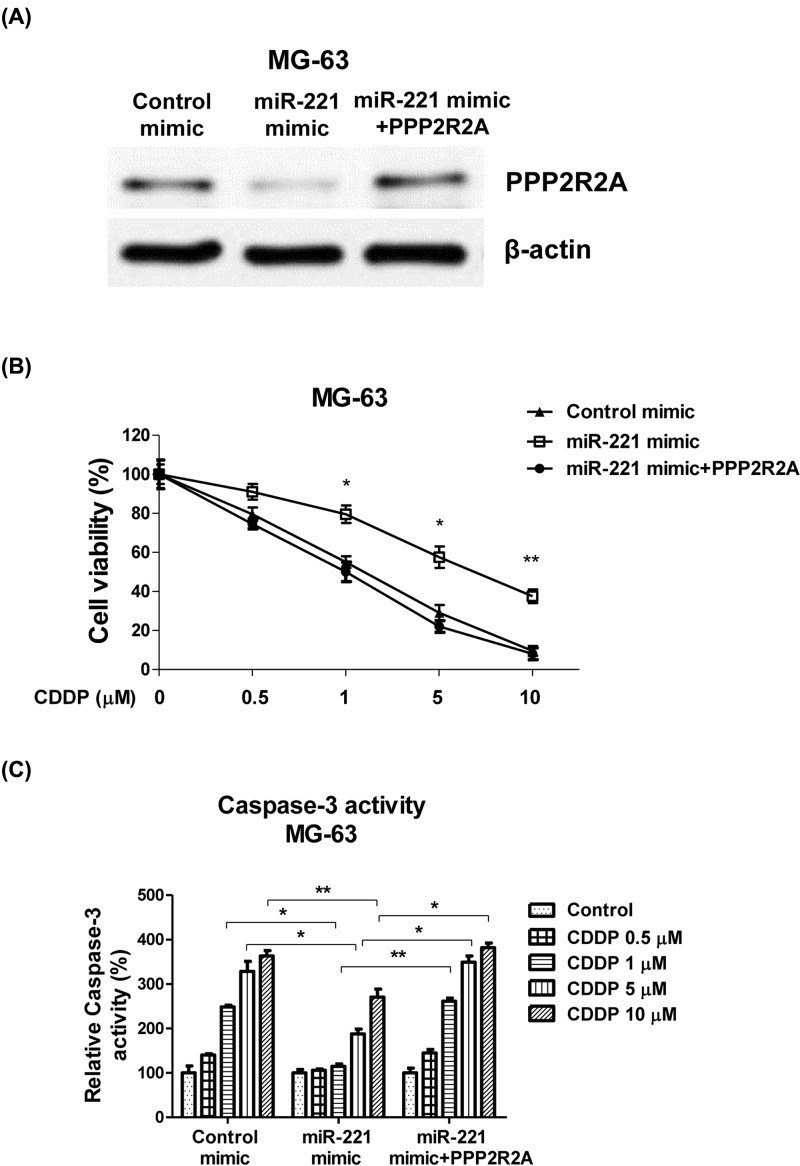
Restoration of PPP2R2A in miR-221-overexpressing cells re-sensitizes OS cells to cisplatin (**A**) MG-63 and SaoS-2 cells were transfected with control mimics, miR-221 mimics, or miR-221 mimics + PPP2R2A for 48 h, followed by Western blot analysis of PPP2R2A expression. (**B**) The above cells were then treated with cisplatin at 0, 0.5, 1, 5, or 10 μM for 48 h. Cell viability was measured via MTT assay and (**C**) cell apoptosis was measured via caspase activity assay. Data are presented as mean ± SD. Columns, mean of three independent experiments; bars, SD; *, *P*<0.05; **, *P*<0.01.

As a tumor suppressor gene, PPP2R2A is reported to be involved in cancer cell proliferation and migration [14]. Since we already demonstrated that miR-221 directly targets PPP2R2A by binding to its 3′-UTR (Figure 4), we hypothesized that the miR-221-modulated cisplatin sensitivity also occurred by directly targeting PPP2R2A. To test this, we ectopically expressed PPP2R2A by transfecting cDNA that only contained the PPP2R2A open reading frame without the 3′-UTR. We co-transfected the PPP2R2A-overexpressing vector along with the miR-221 mimics into MG-63 cells to recover the PPP2R2A expression (Figure 5A). PPP2R2A-restored cells were then treated with 0, 0.5, 1, 5, or 10 μM of cisplatin. As expected, both cell viability and apoptosis assays demonstrated that PPP2R2A restoration recovered the cisplatin sensitivity of the miR-221-overexpressing MG-63 cells (Figure 5B,C). These findings suggest that miR-221 plays important roles in the chemosensitivity of OS cells by directly targeting PPP2R2A.

## Discussion

OS is the most common malignant bone tumor in children and young adults. Despite the combination of surgery and multi-agent chemotherapy, patients with OS who develop resistance to chemotherapy or experience recurrence have a dismal prognosis. Therefore, identification of new biomarkers for OS chemoresistance may limit tumor burden and improve prognosis in patients with OS. Consistent with other studies that reported the up-regulation of miR-221 in multiple cancers [7–11], our results also revealed that miR-221 was up-regulated in cell lines and specimens of patients with OS. The present study demonstrated that miR-221 overexpression promoted the proliferation, migration, and invasion of OS cancer cells *in vitro*. Moreover, we found miR-221 overexpression also attenuated cisplatin-induced cell death in OS cells. A similar study reported that miR-221 knockdown inhibited gastric cancer cell growth and promoted radiosensitivity in gastric carcinoma cells [25]. Cumulatively, these results showed miR-221 to be an oncogenic miRNA in OS.

Cisplatin, a first-line chemotherapeutic agent, is widely used to treat various cancers including OS. However, side effects such as peripheral neuropathy and acquired chemoresistance are major clinical obstacles during therapy [26]. Therefore, enhancing the cisplatin sensitivity of OS cells is an important strategy for effective anti-cancer therapy. miRNAs are reported to be novel modulators of cisplatin sensitivity. Previously, Sun et al. [27] found dysregulated miR-200b to be associated with chemotherapeutic resistance in tongue squamous cell carcinoma (TSCC) cell lines. Moreover, recent studies revealed that miR-221 induced cisplatin resistance in OS cells via the PI3K/Akt pathway [11], which was consistent with our conclusions. However, we described a novel target of miR-221 during the regulation of cisplatin resistance.

PPP2R2A belongs to the PP2A regulatory subunit B family and is a component of the heterotrimeric protein Ser/Thr phosphatase. PP2A activity is down-regulated in cancers, resulting in the activation of various kinases related to proliferation, invasion, and chemoresistance [12]. In this study, we performed bioinformatics analysis and found that PPP2R2A was a potential target of miR-221. We further confirmed that miR-221 directly targeted PPP2R2A in OS cells using western blot and dual-luciferase assays, suggesting miR-221 inhibition to be a potential strategy in developing anti-cancer agents. Our current work demonstrated that miR-221 promoted OS cell proliferation, migration, invasion, and cisplatin resistance by directly targeting PPP2R2A. Restoration of PPP2R2A in miR-221-overexpressing OS cells recovered cisplatin resistance. The precise mechanisms for the miR-221-modulated PPP2R2A down-regulation and the cisplatin resistance are under our further investigation. Studies showed PPP2R2A was an upstream negative effector of Akt/mTOR pathway [18], suggesting down-regulation of PPP2R2A by miR-221 could impair cisplatin sensitivity through the oncogenic Akt pathway. We are working on an *in vivo* xenograft mouse model to evaluate the synergistically inhibitory effects by injecting miR-221 lentivirus and cisplatin into the OS cell-derived xenograft tumors. In addition, recent study demonstrated an *in silico* method to assess the pharmaceutical potential of chemotherapeutic agents to facilitate cytotoxic chemotherapies [28], intriguing us to analyze the drug synergism effects of the combination of miR-221 with cisplatin on human OS tumors using both *in vivo* and *in silico* methods. In general, the present study suggests a new approach by inhibiting miR-221 for anti-chemoresistance in human OS, contributing for the development of new therapeutic drugs.

## Supporting information

**Supplementary Figure S1:** 

**Supplementary Figure S2:** 

**Supplementary Figure S3:** 

## Abbreviations

FBS: fetal bovine serum
HOb: human osteoblast
miRNA: microRNA
MTT: 3-(4,5-dimethylthiazol-2-yl)-2,5-diphenyltetrazolium bromide
OS: osteosarcoma
PP2A: protein phosphatase 2
PPP2R2A: PP2A subunit B
qRT-PCR: Reverse Transcription-Polymerase Chain Reaction
RIPA: Radio-Immunoprecipitation Assay
Ser/Thr: serine/threonine
