# Correction: MicroRNA-221 promotes cisplatin resistance in osteosarcoma cells by targeting PPP2R2A

**DOI:** 10.1042/BSR-2019-0198_COR

**Published:** 2022-01-18

**Keywords:** cisplatin, microRNA-221, osteosarcoma cells

The authors of the original article “MicroRNA-221 promotes cisplatin resistance in osteosarcoma cells by targeting PPP2R2A” (*Biosci Rep* (2019) **39**(7), https://doi.org/10.1042/BSR20190198) would like to correct Figure 2D, as they had placed an incorrect image during the figure build of their submitted article. A revised version of Figure 2 is present in this Correction. The authors express their sincere apologies for any inconvenience that this error has caused to the readers.

**Figure 2:**
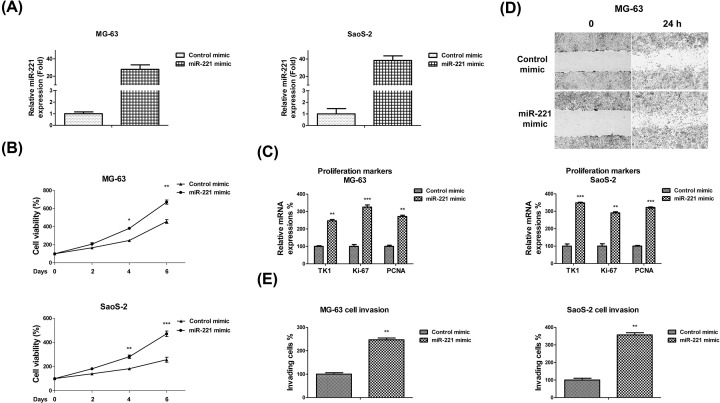
miR-221 overexpression promotes OS cell proliferation, migration, and invasion (**A**) miR-221 or control mimics were transfected into MG-63 (left) and SaoS-2 (right) cells at 50 nM concentrations for 48 h. miR-221 expressions were analyzed using qRT-PCR and normalized to RNU6. (**B**) MG-63 (upper) and SaoS-2 (lower) cells were transfected with 50 nM of control or miR-221 mimics for 48 h, followed by the measurements of cell proliferation via MTT assay and (**C**) measurements of the cell proliferation markers, TK1, Ki-67, and PCNA, using qRT-PCR. (**D**) MG-63 cells were transfected with 50 nM of control or miR-221 mimics for 48 h. Cell migration was measured via wound healing assay and (**E**) cell invasion was measured via transwell assay. Data are presented as mean ± SD. Columns, mean of three independent experiments; bars, SD; *, *P*<0.05; **, *P*<0.01; ***, *P*<0.001.

