# Supplementary figures and images for: MicroRNA-221 promotes cisplatin resistance in osteosarcoma cells by targeting PPP2R2A
Source: Biosci Rep. 2019 Jul 10;39(7):BSR20190198. doi: 10.1042/BSR20190198 (PMC6620383; doi:10.1042/BSR20190198)

## miR-221 Expressions

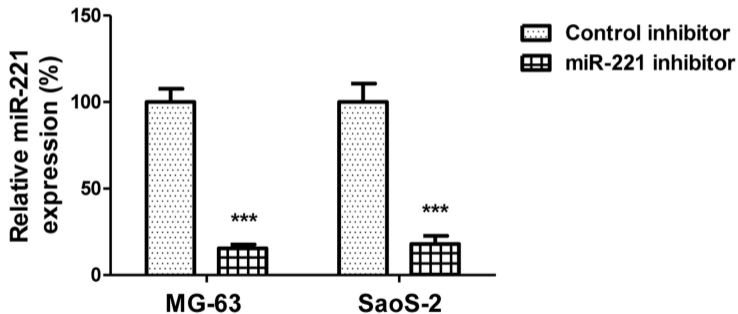

**A**

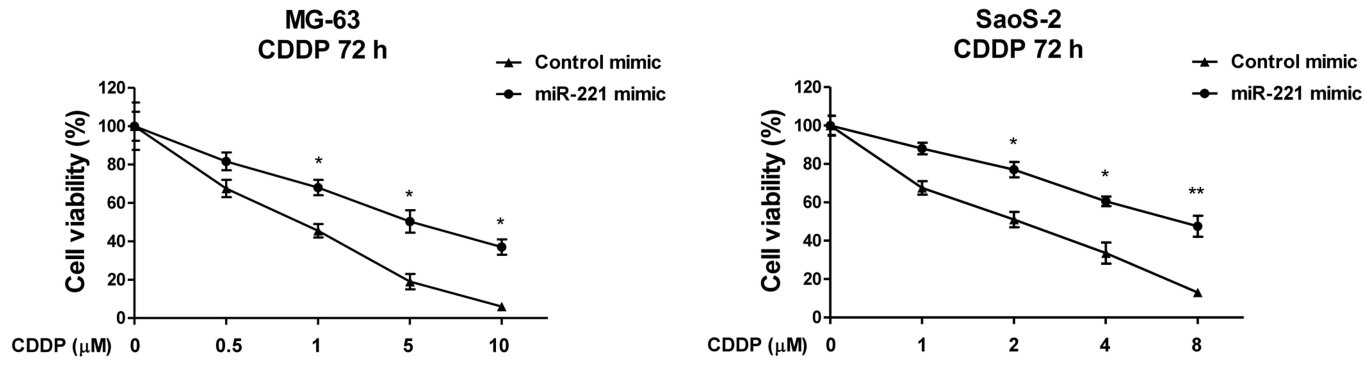

**B**

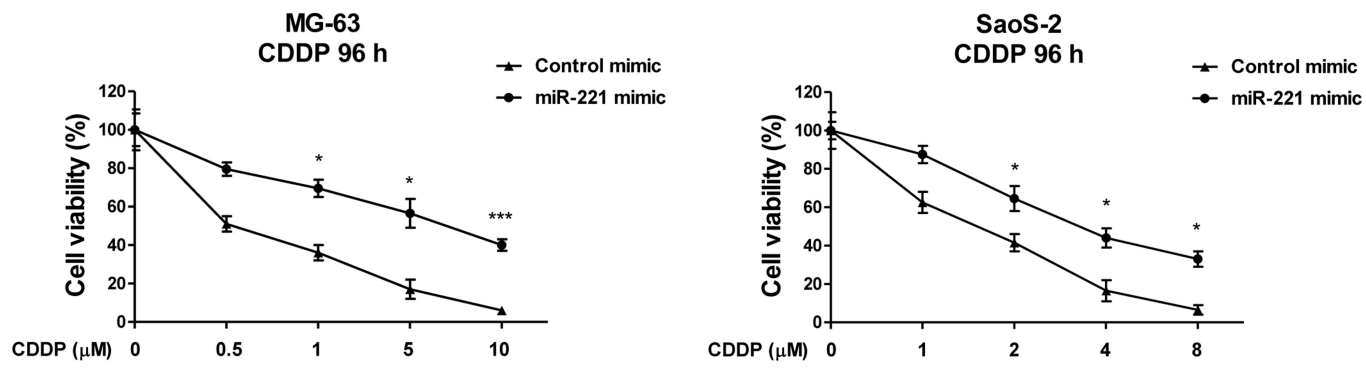

**C**

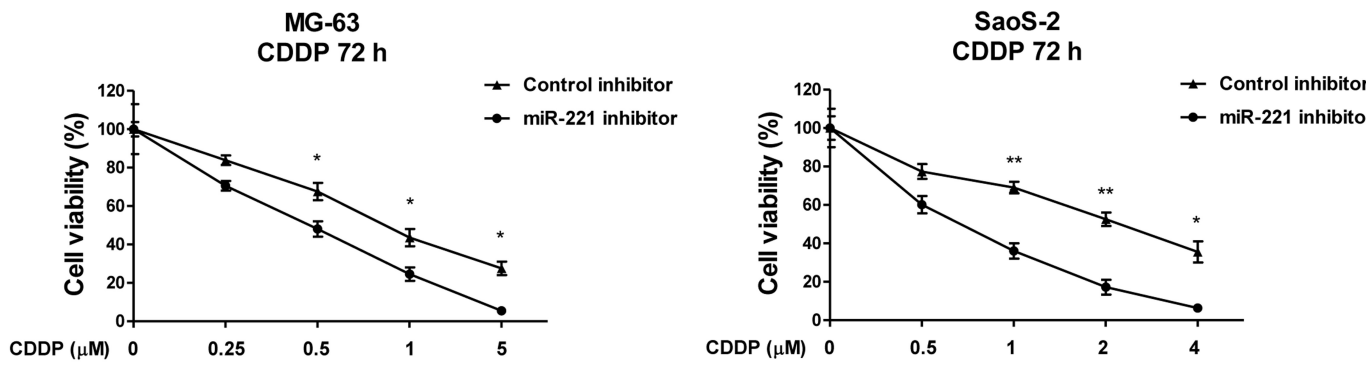

**D**

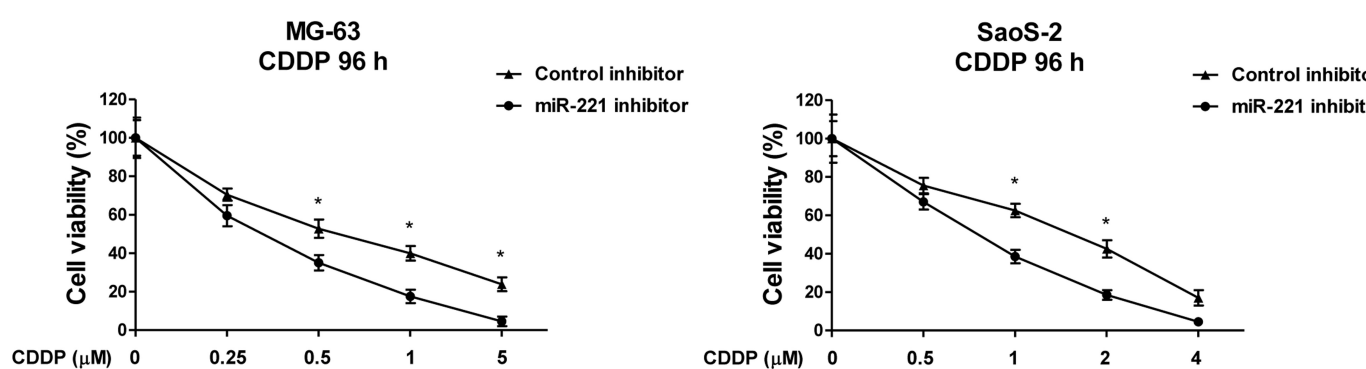

**A**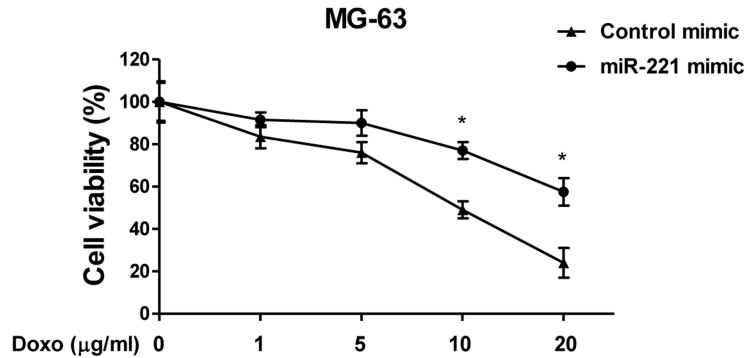**B**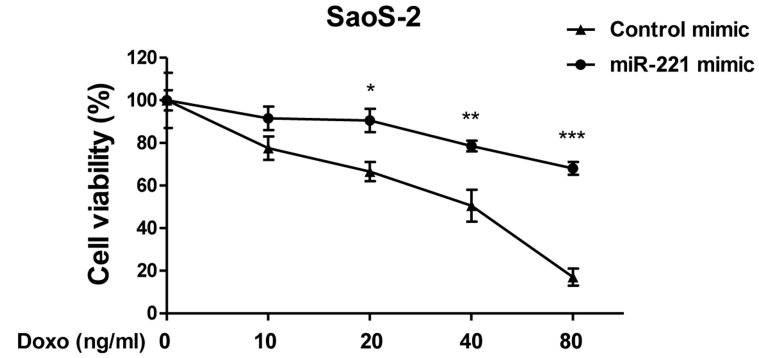**C**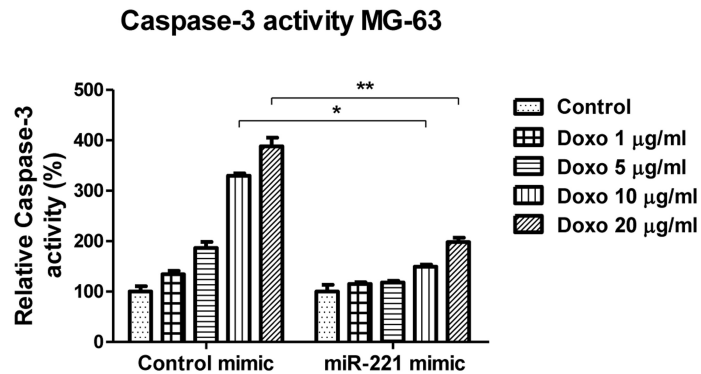**D**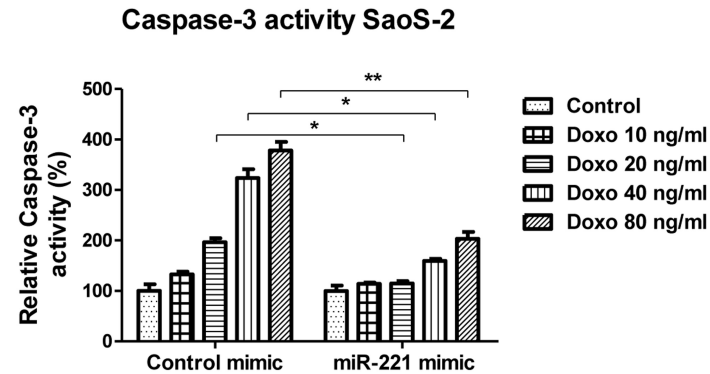

Supplement: Supplementary file 1 [file bsr20190198_Supp1.pdf]
